# Tetra­aqua­bis(nicotinamide-κ*N* ^1^)nickel(II) bis­(2-fluoro­benzoate)

**DOI:** 10.1107/S1600536809040392

**Published:** 2009-10-10

**Authors:** Tuncer Hökelek, Hakan Dal, Barış Tercan, F. Elif Özbek, Hacali Necefoğlu

**Affiliations:** a Department of Physics, Hacettepe University, 06800 Beytepe, Ankara, Turkey; b Department of Chemistry, Faculty of Science, Anadolu University, 26470 Yenibağlar, Eskişehir, Turkey; c Department of Physics, Karabük University, 78050 Karabük, Turkey; d Department of Chemistry, Kafkas University, 63100 Kars, Turkey

## Abstract

The asymmetric unit of the title complex, [Ni(C_6_H_6_N_2_O)_2_(H_2_O)_4_](C_7_H_4_FO_2_)_2_, contains one-half of the complex cation with the Ni^II^ atom located on an inversion center, and a 2-fluoro­benzoate (FB) counter-anion. The four O atoms in the equatorial plane around the Ni atom form a slightly distorted square-planar arrangement with an average Ni—O bond length of 2.079 Å, and the slightly distorted octa­hedral coordination is completed by the two N atoms of the nicotinamide (NA) ligands in the axial positions. The dihedral angle between the carboxyl group and the attached benzene ring is 28.28 (11)°, while the pyridine and benzene rings are oriented at a dihedral angle of 8.31 (4)°. In the crystal structure, O—H⋯O, N—H⋯O, C—H⋯O, and C—H⋯F hydrogen bonds link the mol­ecules into a three-dimensional network. π–π Contacts between the pyridine and benzene rings [centroid–centroid distance = 3.626 (1) Å] may further stabilize the crystal structure. The 2-fluoro­benzoate anion is disordered over two orientations, with an occupancy ratio of 0.85:0.15.

## Related literature

For niacin, see: Krishnamachari (1974 ▶) and for the nicotinic acid derivative *N*,*N*-diethyl­nicotinamide, see: Bigoli *et al.* (1972 ▶). For related structures, see: Hökelek *et al.* (2009 ▶); Sertçelik *et al.* (2009 ▶).
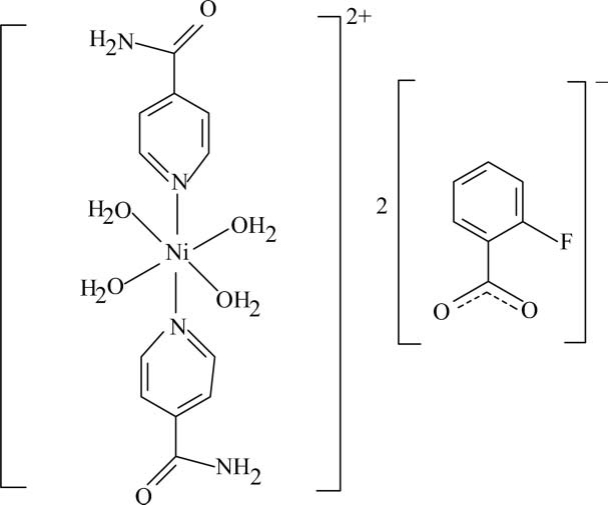


## Experimental

### 

#### Crystal data

[Ni(C_6_H_6_N_2_O)_2_(H_2_O)_4_](C_7_H_4_FO_2_)_2_
*M* *_r_* = 653.23
Triclinic, 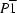

*a* = 7.2529 (1) Å
*b* = 7.3315 (1) Å
*c* = 14.3831 (3) Å
α = 82.115 (2)°
β = 77.332 (2)°
γ = 63.664 (1)°
*V* = 668.05 (2) Å^3^
*Z* = 1
Mo *K*α radiation
μ = 0.81 mm^−1^
*T* = 100 K
0.33 × 0.28 × 0.18 mm

#### Data collection

Bruker Kappa APEXII CCD area-detector diffractometer
Absorption correction: multi-scan (*SADABS*; Bruker, 2005 ▶) *T*_min_ = 0.768, *T*_max_ = 0.868
12196 measured reflections
3339 independent reflections
3241 reflections with *I* > 2σ(*I*)
*R*_int_ = 0.020

#### Refinement

*R*[*F* ^2^ > 2σ(*F* ^2^)] = 0.026
*wR*(*F* ^2^) = 0.071
*S* = 1.04
3339 reflections
221 parameters
7 restraints
H atoms treated by a mixture of independent and constrained refinement
Δρ_max_ = 0.55 e Å^−3^
Δρ_min_ = −0.70 e Å^−3^

### 

Data collection: *APEX2* (Bruker, 2007 ▶); cell refinement: *SAINT* (Bruker, 2007 ▶); data reduction: *SAINT*; program(s) used to solve structure: *SHELXS97* (Sheldrick, 2008 ▶); program(s) used to refine structure: *SHELXL97* (Sheldrick, 2008 ▶); molecular graphics: *ORTEP-3 for Windows* (Farrugia, 1997 ▶); software used to prepare material for publication: *WinGX* (Farrugia, 1999 ▶) and *PLATON* (Spek, 2009 ▶).

## Supplementary Material

Crystal structure: contains datablocks I, global. DOI: 10.1107/S1600536809040392/xu2610sup1.cif

Structure factors: contains datablocks I. DOI: 10.1107/S1600536809040392/xu2610Isup2.hkl

Additional supplementary materials:  crystallographic information; 3D view; checkCIF report

## Figures and Tables

**Table 1:** Selected bond lengths (Å)

Ni1—O4	2.0925 (10)
Ni1—O5	2.0658 (10)
Ni1—N1	2.0834 (11)

**Table 2:** Hydrogen-bond geometry (Å, °)

*D*—H⋯*A*	*D*—H	H⋯*A*	*D*⋯*A*	*D*—H⋯*A*
N2—H2*A*⋯O1^i^	0.86	2.03	2.8875 (17)	171
N2—H2*B*⋯O2^ii^	0.86	2.23	3.0654 (16)	164
O4—H4*A*⋯O3^iii^	0.887 (16)	2.01 (3)	2.8372 (15)	155 (2)
O4—H4*B*⋯O3^iv^	0.887 (16)	1.87 (2)	2.7288 (15)	163 (2)
O5—H5*A*⋯O2^ii^	0.887 (16)	1.82 (2)	2.7001 (15)	175 (3)
O5—H5*B*⋯O3^v^	0.887 (15)	1.94 (2)	2.7774 (15)	157 (2)
C10—H10⋯O2^ii^	0.93	2.52	3.339 (2)	147
C12—H12⋯F1^vi^	0.93	2.51	3.4314 (19)	173

Symmetry codes: (i) 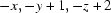; (ii) 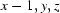; (iii) 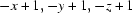; (iv) 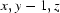; (v) 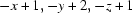; (vi) 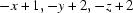.

**Fig. 1.**
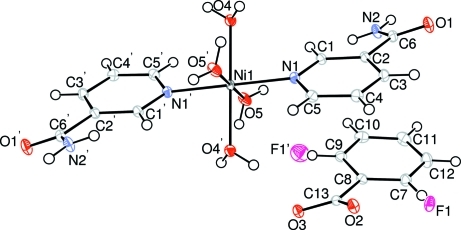
The molecular structure of the title molecule with the atom-numbering scheme. Displacement ellipsoids are drawn at the 50% probability level. Primed atoms are generated by the symmetry operator:(') 1-x, 1-y, 1-z.

## supplementary crystallographic information

### Comment

As a part of our ongoing investigation on transition metal complexes of nicotinamide (NA), one form of niacin (Krishnamachari, 1974), and/or the nicotinic acid derivative *N*,*N*-diethylnicotinamide (DENA), an important respiratory stimulant (Bigoli *et al.*, 1972), the title compound was synthesized and its crystal structure is reported herein.

The title compound is a monomeric complex, with Ni^II^ ion on a centre of symmetry, consisting of two NA ligands, four coordinated water molecules and one FB molecule. The structures of some DENA and/or NA complexes of Ni^II^ ion, [Ni(C_6_H_6_N_2_O)_2_(H_2_O)_4_](C_8_H_5_O_3_)_2_.2H_2_O (Hökelek *et al.*, 2009) and [Ni(C_8_H_5_O_3_)_2_(C_10_H_14_N_2_O)_2_(H_2_O)_2_] (Sertçelik *et al.*, 2009) have also been determined.

In the title compound, NA ligands are monodentate. The four O atoms (O4, O5, and the symmetry-related atoms, O4', O5') in the equatorial plane around the Ni atom form a slightly distorted square-planar arrangement, while the slightly distorted octahedral coordination is completed by the two pyridine N atoms (N1, N1') of the NA ligands at 2.0834 (11) Å from the Ni atom in the axial positions (Table 1, Fig. 1). The average Ni—O bond length is 2.0792 (10) Å. The dihedral angle between the planar carboxylate group (O2/O3/C13) and the benzene ring B (C7—C12) is 28.28 (11)°, while that between rings A (N1/C1—C5) and B is 8.31 (4)°. In the crystal structure, O—H···O, N—H···O, C—H···O and C—H···F hydrogen bonds (Table 2) link the molecules into a three-dimensional network, in which they may be effective in the stabilization of the structure. The π–π contact between the pyridine and benzene rings, *Cg*1—*Cg*2, [where *Cg*1 and *Cg*2 are centroids of the rings A (N1/C1—C5) and B (C7—C12), respectively] may further stabilize the structure, with centroid-centroid distance of 3.626 (1) Å.

### Experimental

The title compound was prepared by the reaction of NiSO_4_.6H_2_O (1.31 g, 5 mmol) in H_2_O (20 ml) and NA (1.22 g, 10 mmol) in H_2_O (20 ml) with sodium 2-fluorobenzoate (1.62 g, 10 mmol) in H_2_O (50 ml). The mixture was filtered and set aside to crystallize at ambient temperature for five days, giving blue single crystals.

### Refinement

Atoms H4A, H4B, H5A and H5B (for H_2_O) were located in difference Fourier map and refined isotropically, with restrains of O4—H4A = 0.887 (16), O4—H4B = 0.887 (16), O5—H5A = 0.887 (16), O5—H5B = 0.887 (15) and H4A—O4—H4B = 106 (2), H5A—O5—H5B = 106 (2)°. The remaining H atoms were positioned geometrically with N—H = 0.86 Å (for NH_2_) and C—H = 0.93 Å for aromatic H atoms and constrained to ride on their parent atoms, with *U*_iso_(H) = 1.2*U*_eq_(C,N). The F1 and H9 atoms attached at C7 and C9, respectively, are disordered over two orientations. During the refinement process, the disordered F1, H9 and F1', H9' atoms were refined with occupancies of 0.85 and 0.15, respectively.

### Crystal data

**Table:**

[Ni(C_6_H_6_N_2_O)_2_(H_2_O)_4_](C_7_H_4_FO_2_)_2_	*Z* = 1
*M**_r_* = 653.23	*F*(000) = 338
Triclinic, *P*1	*D*_x_ = 1.624 Mg m^−^^3^
Hall symbol: -P 1	Mo *K*α radiation, λ = 0.71073 Å
*a* = 7.2529 (1) Å	Cell parameters from 9281 reflections
*b* = 7.3315 (1) Å	θ = 2.9–28.5°
*c* = 14.3831 (3) Å	µ = 0.81 mm^−^^1^
α = 82.115 (2)°	*T* = 100 K
β = 77.332 (2)°	Block, blue
γ = 63.664 (1)°	0.33 × 0.28 × 0.18 mm
*V* = 668.05 (2) Å^3^	

### Data collection

**Table:**

Bruker Kappa APEXII CCD area-detector diffractometer	3339 independent reflections
Radiation source: fine-focus sealed tube	3241 reflections with *I* > 2σ(*I*)
graphite	*R*_int_ = 0.020
φ and ω scans	θ_max_ = 28.5°, θ_min_ = 1.5°
Absorption correction: multi-scan (*SADABS*; Bruker, 2005)	*h* = −9→9
*T*_min_ = 0.768, *T*_max_ = 0.868	*k* = −9→9
12196 measured reflections	*l* = −19→18

### Refinement

**Table:**

Refinement on *F*^2^	Primary atom site location: structure-invariant direct methods
Least-squares matrix: full	Secondary atom site location: difference Fourier map
*R*[*F*^2^ > 2σ(*F*^2^)] = 0.026	Hydrogen site location: inferred from neighbouring sites
*wR*(*F*^2^) = 0.071	H atoms treated by a mixture of independent and constrained refinement
*S* = 1.04	*w* = 1/[σ^2^(*F*_o_^2^) + (0.0351*P*)^2^ + 0.4742*P*] where *P* = (*F*_o_^2^ + 2*F*_c_^2^)/3
3339 reflections	(Δ/σ)_max_ < 0.001
221 parameters	Δρ_max_ = 0.55 e Å^−^^3^
7 restraints	Δρ_min_ = −0.70 e Å^−^^3^

### Special details

**Table:**

Geometry. All e.s.d.'s (except the e.s.d. in the dihedral angle between two l.s. planes) are estimated using the full covariance matrix. The cell e.s.d.'s are taken into account individually in the estimation of e.s.d.'s in distances, angles and torsion angles; correlations between e.s.d.'s in cell parameters are only used when they are defined by crystal symmetry. An approximate (isotropic) treatment of cell e.s.d.'s is used for estimating e.s.d.'s involving l.s. planes.
Refinement. Refinement of *F*^2^ against ALL reflections. The weighted *R*-factor *wR* and goodness of fit *S* are based on *F*^2^, conventional *R*-factors *R* are based on *F*, with *F* set to zero for negative *F*^2^. The threshold expression of *F*^2^ > σ(*F*^2^) is used only for calculating *R*-factors(gt) *etc*. and is not relevant to the choice of reflections for refinement. *R*-factors based on *F*^2^ are statistically about twice as large as those based on *F*, and *R*- factors based on ALL data will be even larger.

### Fractional atomic coordinates and isotropic or equivalent isotropic displacement parameters (Å^2^)

**Table:**

	*x*	*y*	*z*	*U*_iso_*/*U*_eq_	Occ. (<1)
Ni1	0.5000	0.5000	0.5000	0.00977 (8)	
F1	0.68826 (15)	0.96457 (16)	0.86081 (7)	0.0165 (2)	0.85
F1'	0.3316 (12)	0.9351 (12)	0.6229 (2)	0.0341 (16)	0.15
O1	0.27958 (17)	0.42766 (17)	0.97115 (7)	0.0185 (2)	
O2	0.90572 (16)	0.80284 (16)	0.68605 (7)	0.0151 (2)	
O3	0.70091 (16)	0.95073 (15)	0.57688 (7)	0.0139 (2)	
O4	0.36327 (16)	0.29566 (15)	0.53691 (7)	0.01338 (19)	
H4A	0.317 (4)	0.256 (4)	0.4950 (15)	0.040 (6)*	
H4B	0.457 (3)	0.184 (3)	0.5609 (16)	0.035 (6)*	
O5	0.21175 (16)	0.74407 (16)	0.53176 (7)	0.0165 (2)	
H5A	0.109 (3)	0.771 (4)	0.5817 (14)	0.038 (6)*	
H5B	0.205 (4)	0.863 (3)	0.5056 (15)	0.032 (6)*	
N1	0.54874 (18)	0.45933 (17)	0.64030 (8)	0.0115 (2)	
N2	0.04456 (19)	0.57587 (19)	0.87183 (8)	0.0159 (2)	
H2A	−0.0596	0.5881	0.9169	0.019*	
H2B	0.0239	0.6181	0.8146	0.019*	
C1	0.3928 (2)	0.4781 (2)	0.71424 (9)	0.0119 (2)	
H1	0.2651	0.4963	0.7017	0.014*	
C2	0.4136 (2)	0.4717 (2)	0.80876 (9)	0.0120 (2)	
C3	0.6051 (2)	0.4424 (2)	0.82775 (10)	0.0138 (3)	
H3	0.6241	0.4368	0.8901	0.017*	
C4	0.7670 (2)	0.4217 (2)	0.75166 (10)	0.0141 (3)	
H4	0.8966	0.4022	0.7623	0.017*	
C5	0.7335 (2)	0.4306 (2)	0.65972 (10)	0.0132 (3)	
H5	0.8432	0.4160	0.6092	0.016*	
C6	0.2383 (2)	0.4915 (2)	0.89077 (10)	0.0134 (3)	
C7	0.5243 (2)	0.9628 (2)	0.83226 (10)	0.0148 (3)	
H9'	0.6417	0.9567	0.8511	0.018*	0.15
C8	0.5354 (2)	0.9368 (2)	0.73699 (9)	0.0116 (2)	
C9	0.3553 (2)	0.9481 (2)	0.71176 (10)	0.0135 (3)	
H9	0.3565	0.9329	0.6485	0.016*	0.85
C10	0.1751 (2)	0.9811 (2)	0.77808 (11)	0.0173 (3)	
H10	0.0579	0.9859	0.7596	0.021*	
C11	0.1708 (2)	1.0070 (2)	0.87229 (11)	0.0204 (3)	
H11	0.0495	1.0310	0.9170	0.025*	
C12	0.3461 (2)	0.9975 (2)	0.90026 (11)	0.0200 (3)	
H12	0.3440	1.0141	0.9635	0.024*	
C13	0.7298 (2)	0.8941 (2)	0.66226 (9)	0.0114 (2)	

### Atomic displacement parameters (Å^2^)

**Table:**

	*U*^11^	*U*^22^	*U*^33^	*U*^12^	*U*^13^	*U*^23^
Ni1	0.00894 (12)	0.01136 (12)	0.00765 (12)	−0.00371 (9)	−0.00012 (8)	−0.00058 (8)
F1	0.0127 (5)	0.0252 (5)	0.0127 (4)	−0.0076 (4)	−0.0042 (4)	−0.0034 (4)
F1'	0.030 (4)	0.037 (4)	0.036 (4)	−0.016 (3)	−0.006 (3)	0.001 (3)
O1	0.0174 (5)	0.0283 (6)	0.0095 (4)	−0.0104 (4)	−0.0026 (4)	0.0024 (4)
O2	0.0109 (5)	0.0206 (5)	0.0122 (4)	−0.0058 (4)	−0.0025 (4)	0.0013 (4)
O3	0.0137 (5)	0.0168 (5)	0.0107 (4)	−0.0064 (4)	−0.0025 (4)	0.0014 (4)
O4	0.0142 (5)	0.0141 (5)	0.0121 (4)	−0.0062 (4)	−0.0033 (4)	0.0006 (4)
O5	0.0129 (5)	0.0147 (5)	0.0149 (5)	−0.0026 (4)	0.0035 (4)	0.0007 (4)
N1	0.0115 (5)	0.0120 (5)	0.0100 (5)	−0.0044 (4)	−0.0012 (4)	−0.0008 (4)
N2	0.0130 (6)	0.0232 (6)	0.0097 (5)	−0.0075 (5)	−0.0005 (4)	0.0017 (4)
C1	0.0112 (6)	0.0132 (6)	0.0108 (6)	−0.0052 (5)	−0.0015 (5)	−0.0001 (5)
C2	0.0131 (6)	0.0120 (6)	0.0100 (6)	−0.0051 (5)	−0.0012 (5)	0.0002 (5)
C3	0.0157 (6)	0.0149 (6)	0.0111 (6)	−0.0063 (5)	−0.0043 (5)	0.0006 (5)
C4	0.0120 (6)	0.0147 (6)	0.0160 (6)	−0.0054 (5)	−0.0046 (5)	0.0004 (5)
C5	0.0109 (6)	0.0137 (6)	0.0136 (6)	−0.0047 (5)	−0.0006 (5)	−0.0003 (5)
C6	0.0147 (6)	0.0156 (6)	0.0104 (6)	−0.0076 (5)	−0.0006 (5)	−0.0013 (5)
C7	0.0141 (6)	0.0165 (6)	0.0142 (6)	−0.0066 (5)	−0.0034 (5)	−0.0004 (5)
C8	0.0104 (6)	0.0108 (6)	0.0119 (6)	−0.0039 (5)	−0.0008 (5)	0.0003 (4)
C9	0.0136 (6)	0.0130 (6)	0.0145 (6)	−0.0059 (5)	−0.0038 (5)	0.0011 (5)
C10	0.0117 (6)	0.0162 (6)	0.0233 (7)	−0.0061 (5)	−0.0027 (5)	0.0010 (5)
C11	0.0147 (7)	0.0224 (7)	0.0194 (7)	−0.0068 (6)	0.0043 (5)	−0.0015 (6)
C12	0.0205 (7)	0.0244 (7)	0.0127 (6)	−0.0086 (6)	0.0010 (5)	−0.0037 (5)
C13	0.0121 (6)	0.0113 (6)	0.0115 (6)	−0.0060 (5)	−0.0010 (5)	−0.0012 (5)

### Geometric parameters (Å, °)

**Table:**

Ni1—O4	2.0925 (10)	C2—C3	1.3913 (19)
Ni1—O4^i^	2.0925 (10)	C2—C6	1.5012 (18)
Ni1—O5	2.0658 (10)	C3—C4	1.3871 (19)
Ni1—O5^i^	2.0658 (10)	C3—H3	0.9300
Ni1—N1	2.0834 (11)	C4—C5	1.3841 (19)
Ni1—N1^i^	2.0834 (11)	C4—H4	0.9300
O1—C6	1.2346 (17)	C5—H5	0.9300
O2—C13	1.2507 (17)	C7—C12	1.383 (2)
O3—C13	1.2728 (16)	C7—C8	1.3894 (19)
O4—H4A	0.887 (16)	C7—H9'	0.9300
O4—H4B	0.887 (16)	C8—C9	1.3963 (19)
O5—H5B	0.887 (16)	C8—C13	1.5088 (18)
O5—H5A	0.887 (15)	C9—C10	1.383 (2)
N1—C1	1.3427 (17)	C9—H9	0.9300
N1—C5	1.3474 (18)	C10—C11	1.386 (2)
N2—C6	1.3350 (18)	C10—H10	0.9300
N2—H2A	0.8600	C11—C12	1.387 (2)
N2—H2B	0.8600	C11—H11	0.9300
C1—C2	1.3924 (18)	C12—H12	0.9300
C1—H1	0.9300		
			
O4—Ni1—O4^i^	180.0	C4—C3—C2	118.59 (12)
O5—Ni1—O4	90.92 (4)	C4—C3—H3	120.7
O5^i^—Ni1—O4	89.08 (4)	C2—C3—H3	120.7
O5—Ni1—O4^i^	89.08 (4)	C5—C4—C3	119.22 (13)
O5^i^—Ni1—O4^i^	90.92 (4)	C5—C4—H4	120.4
O5^i^—Ni1—O5	180.0	C3—C4—H4	120.4
O5^i^—Ni1—N1	87.25 (4)	N1—C5—C4	122.81 (13)
O5—Ni1—N1	92.75 (4)	N1—C5—H5	118.6
O5^i^—Ni1—N1^i^	92.75 (4)	C4—C5—H5	118.6
O5—Ni1—N1^i^	87.25 (4)	O1—C6—N2	123.50 (13)
N1—Ni1—O4	86.98 (4)	O1—C6—C2	119.09 (13)
N1^i^—Ni1—O4	93.02 (4)	N2—C6—C2	117.41 (12)
N1—Ni1—O4^i^	93.02 (4)	C12—C7—C8	122.85 (13)
N1^i^—Ni1—O4^i^	86.98 (4)	C12—C7—H9'	118.6
N1—Ni1—N1^i^	180.000 (1)	C8—C7—H9'	118.6
C1—N1—C5	117.73 (12)	C7—C8—C9	116.63 (12)
C1—N1—Ni1	121.30 (9)	C7—C8—C13	123.60 (12)
C5—N1—Ni1	120.73 (9)	C9—C8—C13	119.76 (12)
C6—N2—H2A	120.0	C10—C9—C8	121.97 (13)
C6—N2—H2B	120.0	C10—C9—H9	119.0
H2A—N2—H2B	120.0	C8—C9—H9	119.0
Ni1—O4—H4A	122.6 (16)	C9—C10—C11	119.44 (14)
Ni1—O4—H4B	106.3 (15)	C9—C10—H10	120.3
H4A—O4—H4B	106 (2)	C11—C10—H10	120.3
Ni1—O5—H5B	115.1 (15)	C10—C11—C12	120.41 (14)
Ni1—O5—H5A	133.2 (15)	C10—C11—H11	119.8
H5B—O5—H5A	106 (2)	C12—C11—H11	119.8
N1—C1—C2	123.02 (12)	C7—C12—C11	118.69 (14)
N1—C1—H1	118.5	C7—C12—H12	120.7
C2—C1—H1	118.5	C11—C12—H12	120.7
C3—C2—C1	118.63 (12)	O2—C13—O3	124.22 (12)
C3—C2—C6	118.92 (12)	O2—C13—C8	119.65 (12)
C1—C2—C6	122.44 (12)	O3—C13—C8	116.10 (12)
			
O5^i^—Ni1—N1—C1	−139.82 (11)	C3—C4—C5—N1	0.2 (2)
O5—Ni1—N1—C1	40.18 (11)	C3—C2—C6—O1	−19.6 (2)
O4—Ni1—N1—C1	−50.59 (10)	C1—C2—C6—O1	159.27 (13)
O4^i^—Ni1—N1—C1	129.41 (10)	C3—C2—C6—N2	161.24 (13)
O5^i^—Ni1—N1—C5	45.93 (11)	C1—C2—C6—N2	−19.8 (2)
O5—Ni1—N1—C5	−134.07 (11)	C12—C7—C8—C9	0.3 (2)
O4—Ni1—N1—C5	135.16 (11)	C12—C7—C8—C13	−178.58 (13)
O4^i^—Ni1—N1—C5	−44.84 (11)	C7—C8—C9—C10	−0.7 (2)
C5—N1—C1—C2	0.8 (2)	C13—C8—C9—C10	178.17 (12)
Ni1—N1—C1—C2	−173.61 (10)	C8—C9—C10—C11	1.0 (2)
N1—C1—C2—C3	−0.7 (2)	C9—C10—C11—C12	−0.8 (2)
N1—C1—C2—C6	−179.57 (12)	C8—C7—C12—C11	−0.1 (2)
C1—C2—C3—C4	0.3 (2)	C10—C11—C12—C7	0.4 (2)
C6—C2—C3—C4	179.22 (12)	C7—C8—C13—O2	28.4 (2)
C2—C3—C4—C5	−0.1 (2)	C9—C8—C13—O2	−150.41 (13)
C1—N1—C5—C4	−0.6 (2)	C7—C8—C13—O3	−153.53 (13)
Ni1—N1—C5—C4	173.85 (10)	C9—C8—C13—O3	27.64 (18)

Symmetry codes: (i) −*x*+1, −*y*+1, −*z*+1.

### Hydrogen-bond geometry (Å, °)

**Table:**

*D*—H···*A*	*D*—H	H···*A*	*D*···*A*	*D*—H···*A*
N2—H2A···O1^ii^	0.86	2.03	2.8875 (17)	171
N2—H2B···O2^iii^	0.86	2.23	3.0654 (16)	164
O4—H4A···O3^i^	0.89 (2)	2.01 (3)	2.8372 (15)	155 (2)
O4—H4B···O3^iv^	0.89 (2)	1.87 (2)	2.7288 (15)	163 (2)
O5—H5A···O2^iii^	0.89 (2)	1.82 (2)	2.7001 (15)	175 (3)
O5—H5B···O3^v^	0.89 (2)	1.94 (2)	2.7774 (15)	157 (2)
C10—H10···O2^iii^	0.93	2.52	3.339 (2)	147
C12—H12···F1^vi^	0.93	2.51	3.4314 (19)	173

Symmetry codes: (ii) −*x*, −*y*+1, −*z*+2; (iii) *x*−1, *y*, *z*; (i) −*x*+1, −*y*+1, −*z*+1; (iv) *x*, *y*−1, *z*; (v) −*x*+1, −*y*+2, −*z*+1; (vi) −*x*+1, −*y*+2, −*z*+2.
